# TUMEURS OCULO-ORBITAIRES AU CENTRE NATIONAL HOSPITALIER UNIVERSITAIRE DE BANGUI (CNHUB), RÉPUBLIQUE CENTRAFRICAINE, EN 2022

**DOI:** 10.48327/mtsi.v3i3.2023.396

**Published:** 2023-07-03

**Authors:** Rodrigue Romuald ELIEN GAGNAN YAN ZAOU TOU, Jean Michel MBAÏKOUA, Jess Elio KOSH KOMBA PALET, Nouhoum GUIROU

**Affiliations:** 1 Service d'ophtalmologie du Centre national hospitalier et universitaire de Bangui (CNHUB), Bangui, République centrafricaine; 2 Service d'oncologie du Complexe hospitalier universitaire pédiatrique de Bangui, République centrafricaine; 3 Centre hospitalier et universitaire - Institut d'ophtalmologie tropicale de l'Afrique (CHU-IOTA), Bamako, Mali

**Keywords:** Épidémiologie, Tumeurs oculaires, Carcinome épidermoïde, Blépharorraphie, Rétinoblastome, Angiome, Énucléation, CNHUB, Bangui, République centrafricaine, Afrique subsaharienne, Epidemiology, Ocular tumors, Squamous cell carcinoma, Blepharorraphy, Retinoblastoma, Angioma, Enucleation, CNHUB, Bangui, Central African Republic, Sub-Saharan Africa

## Abstract

**Introduction:**

Les tumeurs oculo-orbitaires sont fréquentes. Leurs aspects cliniques et histologiques sont multiples. La prise en charge des tumeurs oculo-orbitaires est un véritable défi en Afrique subsaharienne, notamment dans le contexte centrafricain. Le but de cette étude est de contribuer à l'amélioration de la prise en charge des tumeurs oculo-orbitaires au Centre national hospitalier et universitaire de Bangui (CNHUB).

**Méthodologie:**

Il s'est agi d'une étude observationnelle prospective des tumeurs oculo-orbitaires sur 12 mois allant du 1^er^ janvier au 31 décembre 2022 dans le service d'ophtalmologie du CNHUB.

**Résultats:**

Nous avons colligé 97 patients dont l'âge moyen était de 37,5 ans avec des extrêmes allant de 2 ans à 70 ans. Il y avait une prédominance masculine (sex-ratio = 1,8). La quasitotalité des patients résidaient à Bangui (96%). Les sujets âgés de moins de 40 ans étaient les plus atteints (55%). Un peu plus de la moitié des tumeurs étaient bénignes (55%). La douleur oculaire (24%), le larmoiement (22%) et la tuméfaction orbitaire (167%) étaient les principaux signes fonctionnels. Les facteurs de risque retrouvés étaient principalement la sénescence (23%), le diabète (22%), l'infection au VIH (16%) et le couple alcool-tabac (37%). Les modalités thérapeutiques les plus utilisées étaient l'exérèse chirurgicale (68%) et l'énucléation (16%). La chimiothérapie était associée à la chirurgie dans 37% des cas.

**Conclusion:**

Les tumeurs oculo-orbitaires sont fréquentes dans notre contexte. Leurs présentations morpho-histologiques sont diverses. Une approche multidisciplinaire est importante pour une bonne prise en charge.

## Abstract (en)

**Introduction:**

Oculo-orbital tumors are common. Their clinical and histological features are multiple. The management of oculo-orbital tumors is a real challenge in sub-Saharan Africa, especially in our context. The aim of this study was to contribute to the improvement of the management of oculo-orbital tumors at the Bangui Teaching Hospital (CNHUB).

**Methodology:**

This was a prospective, observational study of oculo-orbital tumors over 12 months from January 1 to December 31, 2022 in the ophthalmology department of the CNHUB. All consenting patients with histologically proven tumors of the eyeball and/or its adnexa were included in this study, regardless of age and sex.

The parameters studied were:

- epidemiological: age, sex, occupation, origin;
- clinical: time of consultation, functional signs and associated signs, physical examination signs, risk factors;
- paraclinical: report of the anatomopathological examination of the operating room;
- therapeutic: treatment modalities (chemotherapy, surgery, palliative treatment, radiotherapy).

These data were collected on a pre-established survey form for direct administration. The processing and the production of the results were carried out with Excel and Epi Info 7.0 software.

**Results:**

We included 97 patients with a mean age of 37.5 years, ranging from 2 to 70 years. There was a male predominance (sex-ratio = 1.8). Almost all patients lived in Bangui (96%). Patients under 40 years of age were most commonly affected (55%). The majority of tumors were benign (55%). Ocular pain (24%), lacrimation (22%) and orbital swelling (16%) were the main symptoms. Risk factors were mainly age (23%), diabetes (22%), HIV infection (16%) and alcohol and tobacco use (37%). The most common treatments were surgical removal (68%) and enucleation (16%). Chemotherapy was combined with surgery in 37% of cases.

**Conclusion:**

Oculo-orbital tumors are frequent in our context. Their morpho-histological presentations are varied. A multidisciplinary approach is important for a good management.

## Introduction

Les tumeurs oculo-orbitaires, qu'elles soient bénignes, précancéreuses ou malignes peuvent compromettre les pronostics fonctionnel et/ou vital de la personne affectée [2, 3].

Le diagnostic des tumeurs oculo-orbitaires repose sur la conjonction des arguments cliniques, radiologiques et histologiques [2, 3]. Leurs modalités thérapeutiques sont multiples : selon la nature de la tumeur, il peut s'agir de la chirurgie seule ou en combinaison avec d'autres moyens thérapeutiques (chimiothérapie, cryothérapie, thermothérapie et radiothérapie) [2, 3, 7].

En Afrique de l'Ouest, les auteurs ont trouvé du 1^er^ mars au 31 août 2018 une incidence hospitalière de 26,2 cas de tumeurs oculo-orbitaires [5]. En Afrique centrale, leur fréquence institutionnelle variait de 0,4% à 2% [1, 7, 8].

Dans une étude menée en République centrafricaine [6], les tumeurs oculaires représentaient 13,4% des pathologies du segment postérieur de l'œil observées à l'échographie en 2019. La non-inclusion des tumeurs orbi-to-annexielles de l'œil et la nature descriptive de l'étude mentionnée ont été ses principales limites, qui ont conduit à la réalisation de la présente étude. L'objectif poursuivi était de contribuer à l'amélioration de la prise en charge des tumeurs oculo-orbitaires au CNHUB.

## Méthodologie

Il s'est agi d'une étude observationnelle et prospective des tumeurs de l'œil et des annexes sur une période de 12 mois allant de janvier à décembre 2022 au service d'ophtalmologie du Centre national hospitalier et universitaire de Bangui. Tous les patients consentants et présentant une tumeur du globe oculaire et/ou de ses annexes avec une preuve histologique ont été inclus dans cette étude, sans distinction d'âge et de sexe. Les paramètres étudiés ont été:

- épidémiologiques : âge, sexe, profession, provenance;
- cliniques : délai de consultation, signes fonctionnels et signes associés, signes de l'examen physique, facteurs de risque;
- paracliniques : compte rendu de l'examen anatomopathologique de la pièce opératoire;
- thérapeutiques : modalités du traitement (chimiothérapie, chirurgie, traitement palliatif, radiothérapie).

Ces données ont été recueillies sur une fiche d'enquête pré-établie à administration directe. Le traitement et la production des résultats ont été réalisés avec les logiciels Excel* et Epi Info* 7.0.

## Résultats

Quatre vingt-dix sept tumeurs sur 5 554 consultations soit 1,74% de prévalence.

Il y avait 53 tumeurs bénignes (55%) et 44 tumeurs malignes (45%).

La plupart de nos patients résidaient à Bangui (Fig. 1).

**Figure 1:**
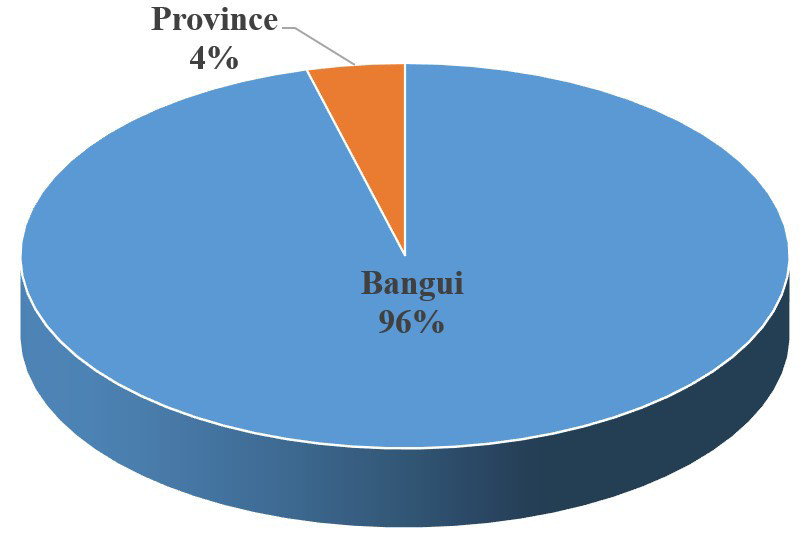
Répartition des patients selon la provenance Distribution of patients according to origin

Le sex-ratio H/F était de 1,8. Les adultes jeunes âgés de moins de 40 ans étaient davantage représentés (55%) (Tableau I). L'âge moyen était de 37,5 ± 1,5 ans avec des extrêmes allant de 2 ans à 70 ans.

**Tableau I:** Relation entre l'âge et le sexe des patients Relationship between age and gender of patients

Sexe	[0 - 10[	[10 - 20[	[20 - 30[	[30 - 40[	[40 - 50[	[50 - 60[	60 et +	Total
**Masculin**	22	3	5	8	14	6	4	62
**Féminin**	4	9	1	1	7	5	8	35
**Total**	26	12	6	9	21	11	12	97

Les principaux motifs de consultations sont exposés dans les tableaux II et III.

**Tableau II:** Répartition des patients en fonction des signes fonctionnels Distribution of patients according to functional signs

Signes fonctionnels	Effectif (n)	Pourcentage (%)
**Douleur**	89	24
**Larmoiement**	81	22
**Tuméfaction orbitaire**	60	16
**Rougeur oculaire**	55	15
**Baisse d'acuité visuelle**	44	12
**Sensation de grains de sable**	42	11

**Tableau III:** Répartition des patients en fonction des signes physiques Distribution of patients according to physical signs

Signes physiques	Effectif (n)	Pourcentage (%)
**Hyperhémie conjonctivale**	75	32
**Sécrétions**	72	30
**Exophtalmie**	43	18
**Excroissance conjonctivale**	42	18
**Tumeur palpébrale**	5	2

Le grand âge (20%), le diabète (19%) et la consommation de tabac et/ou alcool (14%) étaient les facteurs de risque communs aux tumeurs bénignes et malignes (Tableau T4).

**Tableau IV:** Relation entre les facteurs de risque et la nature histologique des tumeurs Table IV: Relationship between risk factors and the histological nature of tumours

Facteurs de risque	Tumeur bénigne	Tumeur maligne	Total
**Âge**	16	7	23
**Diabète**	9	13	22
**VIH**	0	16	16
**Tabac/Alcool**	27	10	37
**Soudeur**	5	0	5
**Absents**	12	0	12

Le ptérygion, tumeur bénigne la plus fréquente, était observé chez les adultes jeunes (30 ans à 50 ans).

Le rétinoblastome, la plus fréquente des tumeurs malignes, se rencontrait chez les enfants de moins de 10 ans (Tableau V).

**Tableau V:** Relation entre le type de la tumeur et l'âge Relationship between type of tumour and age

Tumeurs	[0 - 10[	[10 - 20[	[20 - 30[	[30 - 40[	[40 - 50[	[50 - 60[	60 et +	Total
**Tumeurs bénignes**								
ptérygion	0	0	0	3	16	8	8	35
chalazion	0	3	3	0	0	0	0	6
kyste séreux	0	0	3	1	1	0	0	5
papillome	1	2	0	0	0	0	0	3
**Tumeurs malignes**								
nævus	0	2						
neurofibrome plexiforme	0	2	0	0	0	0	0	2
rétinoblastome	25	0	0	0	0	0	0	25
carcinome épidermoïde	0	0	0	5	4	3	1	13
rhabdomyosarcome	0	2	0	0	0	0	0	2
métastase orbitaire de tumeur de Cavum	0	0	0	0	0	0	2	2
tumeur des VL	0	0	0	0	0	0	1	1
lymphome de Burkitt	0	1	0	0	0	0	0	1
**Total**	**26**	**12**	**6**	**9**	**21**	**11**	**12**	**97**

La localisation préférentielle des tumeurs bénignes était annexielle. Les tumeurs malignes concernaient préférentiellement les structures endo-oculaires (Tableau VI).

**Tableau VI:** Relation entre la localisation anatomique et la nature des tumeurs Relationship between anatomical location and nature of tumours

Localisation	Tumeur bénigne		Tumeur maligne		Total
**Effectif (n)**	**Pourcentage (%)**	**Effectif (n)**	**Pourcentage (%)**
**Paupière**	11	11	0	0	11
**Conjonctive**	42	43	13	13	55
**Orbite**	0	0	6	6	6
**Globe oculaire**	0	0	25	26	25
**Total**	53	55	44	45	97

Nous avons recouru à la chirurgicale radicale dans la quasi-totalité des cas (93,81%).

## Discussion

Au cours de notre étude, si la prise en charge des tumeurs bénignes était aisée, celle des tumeurs malignes était plus difficile. Cette difficulté résultait de l'association de plusieurs facteurs négatifs, à savoir:

- les facteurs liés aux patients (niveau de vie précaire des patients en âge avancé, mauvaise connaissance des patients sur les tumeurs oculaires);
- les facteurs liés à l'offre des soins oculaires (disponibilité de cinq ophtalmologistes mais non formés dans la prise en charge des tumeurs oculo-orbitaires, manque de matériel adéquat pour le traitement conservateur des tumeurs oculo-orbitaires, inexistence des traitements conservateurs de première ligne - par exemple la cryothérapie, la thermothérapie transpupillaire et la brachythérapie);
- les facteurs liés au système de santé : (inaccessibilité géographique du service d'ophtalmologie, inexistence d'un circuit de référencement et de la prise en charge des tumeurs oculo-orbitaires).

Cependant, notre étude est limitée par la présence des biais de sélection, d'information et de recrutement [4] subséquents au recrutement exclusivement hospitalier des patients atteints de tumeurs oculo-orbitaires et consentants. Ces biais pourraient être corrigés par l'échantillonnage probabiliste des patients (randomisation et tirage au sort), la prise en compte de tous les patients, même ceux jugés exempts de tumeurs oculo-orbitaires, et l'inclusion des patients vus dans les centres de santé périphériques et des sujets tirés au sort au sein des communautés. Ensuite, n'étant ni une étude de cohorte, ni une étude castémoin, notre étude ne peut établir les liens de causalités entre les tumeurs oculo-orbitaires et certaines caractéristiques individuelles des patients (âge, sexe, antécédents, terrains, consommation de substances toxiques, etc.).

Nonobstant ces limites, nous pouvons valablement comparer nos résultats avec ceux des travaux antérieurs. Ainsi, le fait que Bangui ait été la ville de provenance de la quasi-totalité de nos patients expliquerait la sous-notification des cas de tumeurs oculo-orbitaires existant dans les provinces. Afin de réduire cette inégalité d'accès aux services de santé oculaire, il est nécessaire de mettre en place une vaste stratégie nationale de dépistage et de prise en charge des tumeurs oculo-orbitaires. Avec l'appui des partenaires au développement, l'élaboration de cette stratégie se fera en neuf étapes:

1. Recrutement des relais communautaires chargés de la sensibilisation, du dépistage et du référencement des cas.
2. Formation des relais communautaires, professionnels de santé, enseignants, journalistes, travailleurs sociaux et forces de sécurité intérieures (agents de la police et de la gendarmerie) sur le dépistage et le référencement des cas.
3. Sensibilisation des leaders politiques, communautaires, religieux et d'opinions sur les tumeurs oculo-orbitaires.
4. Sensibilisation de toute la population (focus group, conférence-débat, presse écrite, médias audiovisuels) sur les tumeurs oculaires (rétinoblastome particulièrement) et leur prise en charge.
5. Formation d'un pool de professionnels de santé oculaire (ophtalmologistes, ocularistes, psychologues) sur les techniques et les principes du traitement conservateur des tumeurs oculaires (notamment le rétinoblastome).
6. Aide au diagnostic des tumeurs oculaires, par la couverture des frais de la tomodensitométrie orbito-cérébrale et de
7. Libération de cases-parents pour l'accueil et le séjour des patients venus des provinces.
8. Amélioration du plateau technique du service d'ophtalmologie du CNHUB pour la réalisation sécurisée des énucléations et de la thermothérapie transpu-pillaire.
9. Facilitation de participation à des forums internationaux pour des communications et l'acquisition des nouvelles expertises du traitement des tumeurs oculaires.

La faiblesse de cette stratégie de dépistage et de prise en charge des tumeurs oculaires, dans notre contexte, et le manque de matériel adéquat pour le traitement conservateur des tumeurs oculo-orbitaires, puis la réticence des parents à l'énucléation, justifieraient la faible fréquence de l'énucléation dans notre contexte. Les raisons du refus parental pour l'énucléation de leur enfant n'ont pas été explorées dans la présente étude.

D'ailleurs, l'énucléation et la chimiothérapie étaient les deux principaux axes thérapeutiques fréquemment utilisés dans notre contexte et dans la sous-région [7, 11]. Le coût onéreux de l'installation des autres alternatives thérapeutiques (radiothérapie, curiethérapie, brachythérapie, etc.), ainsi que l'exigence de leur manipulation par des professionnels de santé qualifiés, les rendent inaccessibles dans notre contexte.

De ce qui précède, nous recommandons:

- à la population : de consulter le plus rapidement possible le centre de santé le plus proche pour toute affection oculaire, notamment le gonflement de tout ou partie de l'œil;
- au personnel de santé : de référer sans délai vers un service de santé oculaire toute affection, surtout les tumeurs oculo-orbitaires;
- aux autorités : de promouvoir une politique de santé oculaire pour tous, avec l'aide des partenaires au développement, par la création d'une stratégie nationale de dépistage et de prise en charge des tumeurs oculo-orbitaires.

## Conclusion

Notre étude a souligné les difficultés de la prise en charge des tumeurs oculo-orbitaires dans le contexte centrafricain. Ces difficultés concernent d'une part les patients et leur entourage, et d'autre part les professionnels de la santé oculaire et le système national de santé. Pour y remédier, il est nécessaire de mettre en place une stratégie nationale de dépistage précoce et de prise en charge des tumeurs oculo-orbitaires.

## Liens d'intérêts

Les auteurs ne déclarent aucun conflit d'intérêts.

## Contribution Des Auteurs

Le Dr Rodrigue ELIEN a collecté les données, opéré les patients et rédigé le manuscrit.

Le Dr Jean Michel MBAÏKOUA a contribué à la prise en charge chirurgicale des patients.

Le Dr Jess Elio KOSH KOMBA PALET a réalisé la chimiothérapie.

Le Pr Nouhoum GUIROU a supervisé la collecte des données et corrigé le manuscrit.

## Iconographie Complémentaire

**Figure 1:**
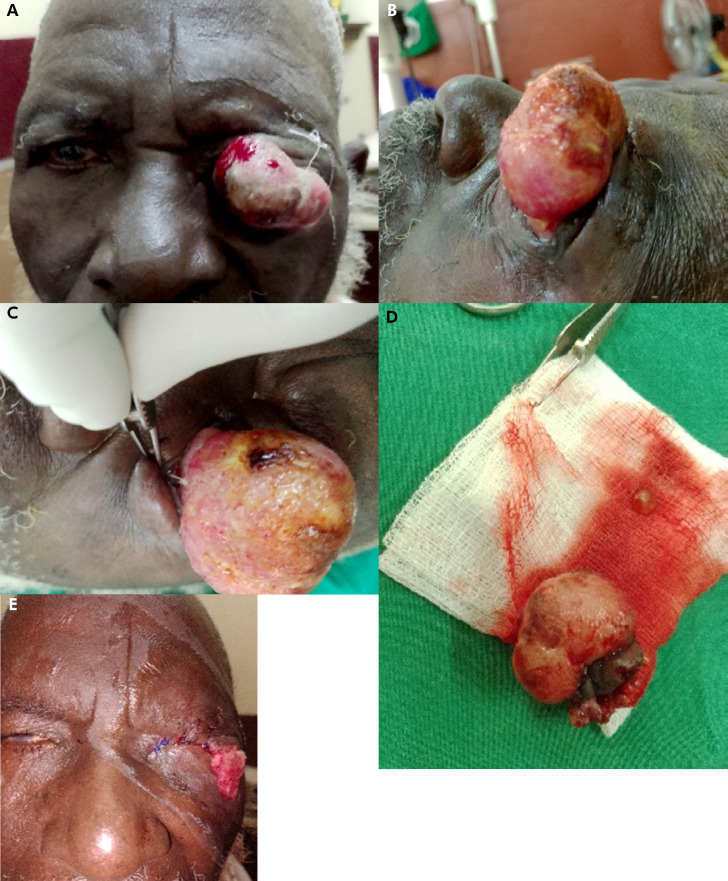
A, B, C - Carcinome épidermoïde conjonctival invasif. D - Pièce opératoire. E - Blépharorraphie gauche avec mise en place d'un drain (mèche de compresse) A, B, C - Invasive conjunctival squamous cell carcinoma. D - Surgical specimen. E - Left blepharorraphy with placement of a drain (wick of gauze)

**Figure 2:**
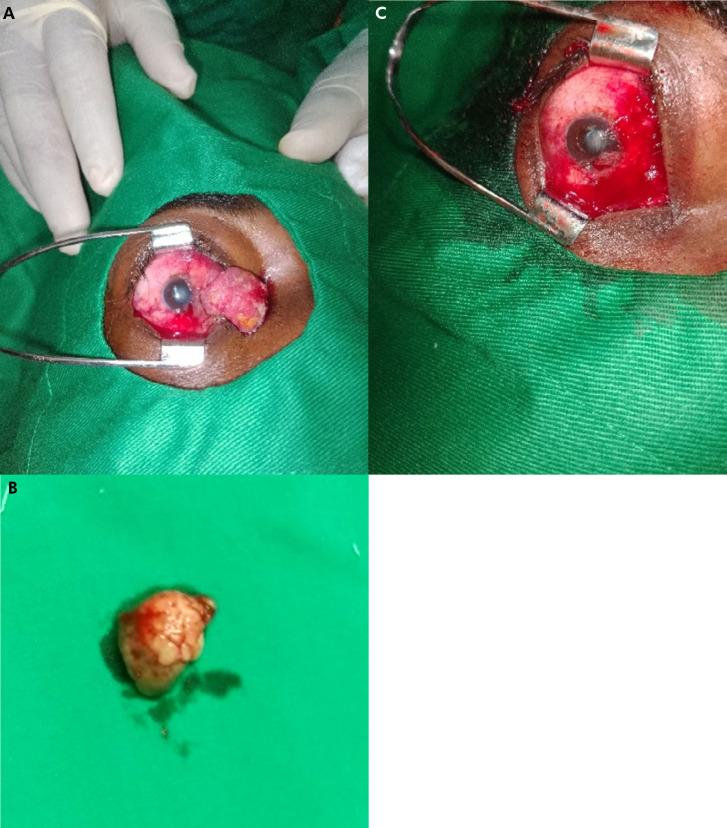
A - Carcinome épidermoïde conjonctival non invasif. B - Pièce opératoire. C - Zone d'exérèse conjonctivale propre A - Non-invasive conjunctival squamous cell carcinoma. B - Surgical specimen. C - Area of clean conjunctival excision

**Figure 3:**
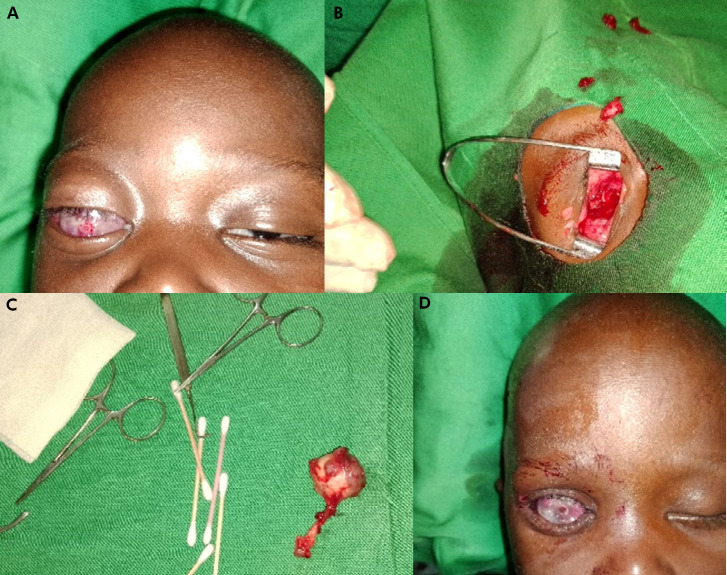
A - Rétinoblastome unilatéral droit groupe E de la classification de l'IRC avant l'énucléation. B - Cavité d'énucléation après section du nerf optique. C - Pièce opératoire (globe oculaire et nerf optique). D - Cavité d'énucléation et mise en place du conformateur A - Right unilateral retinoblastoma group E of the IRC classification before enucleation. B - Enucleation cavity after section of the optic nerve. C - Surgical specimen (eyeball and optic nerve). D - Enucleation cavity and placement of the conformer

**Figure 4:**
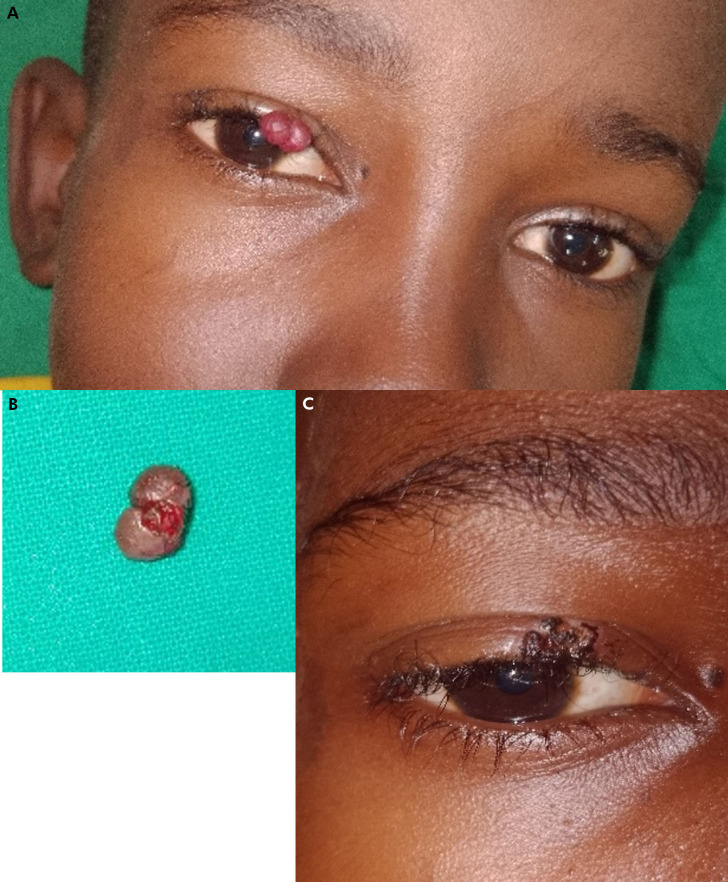
A - Angiome palpébral polylobé de la paupière supérieure droite. B - Pièce opératoire. C - Cicatrice de la zone d'exérèse de la paupière supérieure droite A - Polylobed palpebral angioma of the right upper eyelid. B - Surgical specimen. C - Scar in the excision zone of the right upper eyelid

**Figure 5:**
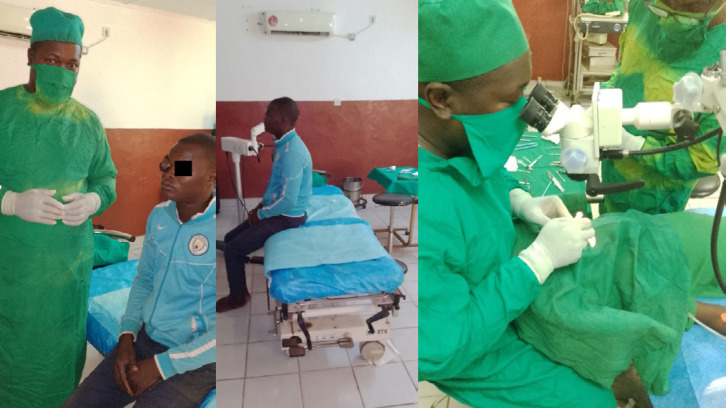
Salle opératoire du CNHUB avec plateau technique obsolète CNHUB operating theatre with obsolete technical equipment

**Figure 6:**
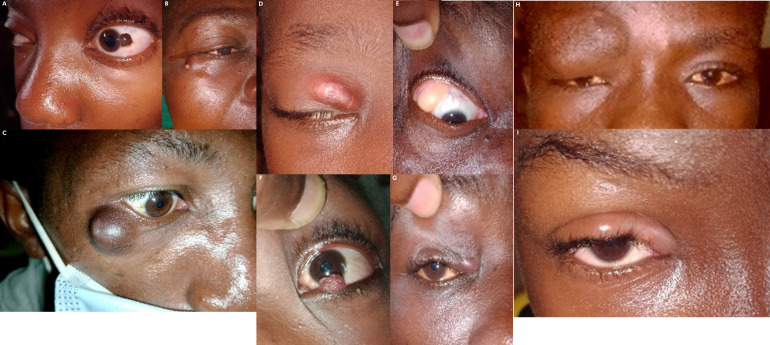
A - Naevus conjonctival. B - Kyste palpébral. C-D - Lipome palpébral. E - Localisation ectopique de la glande lacrymale principale. F - Papillome palpébrale. G- Neurofibrome palpébral (Neurofibromatose de type I). H-I - Orgelet A - Conjunctival nevus. B - Palpebral cyst. C-D - Palpebral lipoma. E - Ectopic location of the main lacrimal gland. F - Palpebral papilloma. G - Palpebral neurofibroma (Neurofibromatosis type I). H-I - Stye

**Figure 7:**
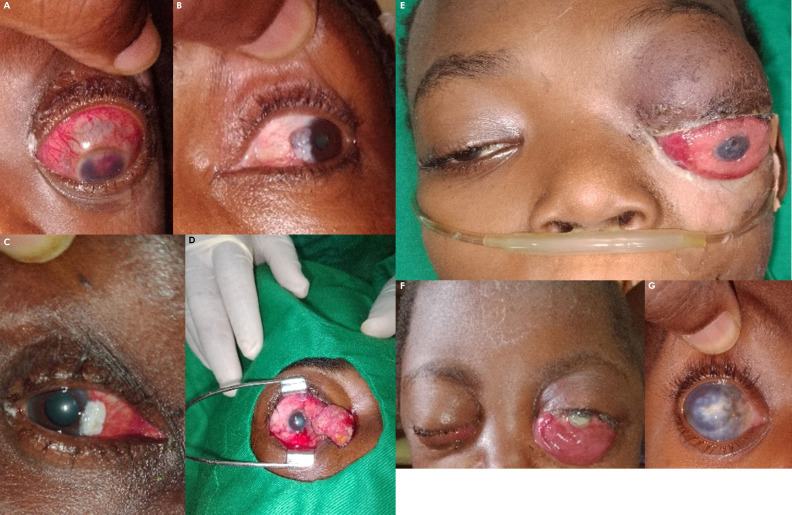
A - Glaucome néovasculaire au cours de rétinoblastome unilatéral stade avancé. B-C - Carcinome épidermoïde conjonctival in situ. D - Carcinome épidermoïde conjonctival invasif. E - Rétinoblastome unilatéral gauche extra-oculaire évolué (stade cM1). F - Métastases oculaires au cours de lymphome de Burkitt. G - Buphtalmie au cours du rétinoblastome unilatéral droit. A - Neovascular glaucoma in advanced unilateral retinoblastoma. B-C - Conjunctival squamous cell carcinoma in situ. D - Invasive conjunctival squamous cell carcinoma. E - Advanced left unilateral extraocular retinoblastoma (stage cM1). F - Ocular metastases in Burkitt's lymphoma. G - Buphthalmia in right unilateral retinoblastoma.

**Figure 8:**
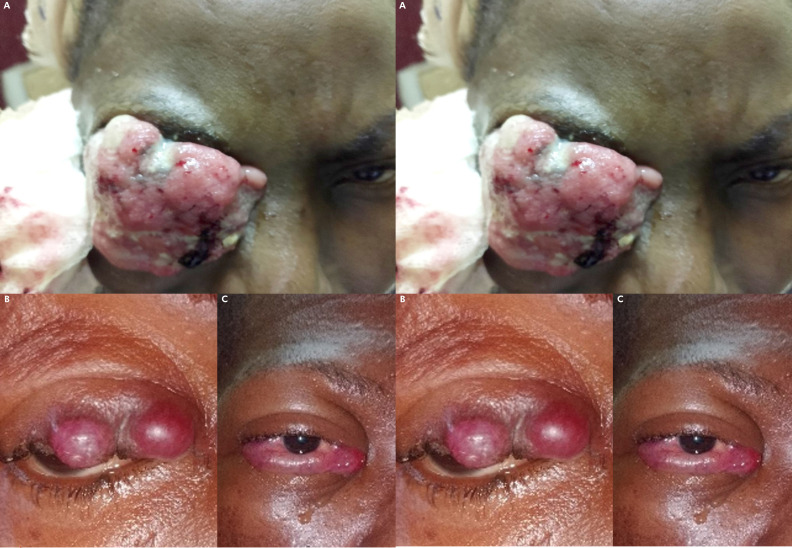
A - Carcinome épidermoïde conjonctivale invasif. B - Hémangiome palpébrale. C - Lymphome orbitaire droit. D - Carcinome conjonctival in situ. E - Metatastase orbitaire bilatérale de cancer du cavum. F - Adénocarcinome du sac lacrymal gauche. A - Invasive conjunctival squamous cell carcinoma. B - Palpebral haemangioma. C - Right orbital lymphoma. D - Conjunctival carcinoma in situ. E - Bilateral orbital metatastasis of cavitary cancer. F - Adenocarcinoma of the left lacrimal sac
